# Rapid Screening of *Mentha spicata* Essential Oil and L-Menthol in *Mentha piperita* Essential Oil by ATR-FTIR Spectroscopy Coupled with Multivariate Analyses

**DOI:** 10.3390/foods10020202

**Published:** 2021-01-20

**Authors:** Osman Taylan, Nur Cebi, Osman Sagdic

**Affiliations:** 1 Department of Industrial Engineering, Faculty of Engineering, King Abdulaziz University, Jeddah 21589, Saudi Arabia; otaylan@kau.edu.sa; 2 Department of Food Engineering, Faculty of Chemical and Metallurgical Engineering, Yıldız Technical University, İstanbul 34210, Turkey; sagdic@gmail.com

**Keywords:** ATR-FTIR, *Mentha piperita* essential oil, PLSR, PCR, HCA, adulteration, chemometrics

## Abstract

*Mentha piperita* essential oil (EO) has high economic importance because of its wide usage area and health-beneficial properties. Besides health-beneficial properties, *Mentha piperita* EO has great importance in the flavor and food industries because of its unique sensory and quality properties. High-valued essential oils are prone to being adulterated with economic motivations. This kind of adulteration deteriorates the quality of authentic essential oil, injures the consumers, and causes negative effects on the whole supply chain from producer to the consumer. The current research used fast, economic, robust, reliable, and effective ATR-FTIR spectroscopy coupled chemometrics of hierarchical cluster analysis(HCA), principal component analysis (PCA), partial least squares regression (PLSR) and principal component regression (PCR) for monitoring of *Mentha spicata* EO and L-menthol adulteration in *Mentha piperita* EOs. Adulterant contents (*Mentha spicata* and L-menthol) were successfully calculated using PLSR and PCR models. Standard error of the cross-validation SECV values changed between 0.06 and 2.14. Additionally, bias and press values showed alteration between 0.06 and1.43 and 0.03 and 41.15, respectively. Authentic *Mentha piperita* was successfully distinguished from adulterated samples, *Mentha spicata* and L-menthol, by HCA and PCA analysis. The results showed that attenuated total reflectance-Fourier transform infrared (ATR-FTIR) spectroscopy, coupled with chemometrics could be effectively used for monitoring various adulterants in essential oils.

## 1. Introduction

*Mentha piperita* belongs to the Lamiaceae family, is one of the most well-known and most utilized herbs throughout the world [1]. *Mentha piperita* is generally defined as peppermint, which is involved in numerous products manufactured in various industries such as flavor, fragrances, cosmetics, aromatherapy, and phytomedicine [2]. According to the international standard, *Mentha piperita* oil is defined as an essential oil which is obtained from the plant *Mentha piperita* by steam distillation of the aerial parts of the plant [3]. *Mentha piperita* EO has high economic importance because of its wide usage area and health-beneficial properties. A number of studies have reported that *Mentha piperita*, medicinal plant showed anti-inflammatory, analgesic, anti-fungal, antimicrobial, and central nervous system excitation effects [4].

Besides health-beneficial properties, *Mentha piperita* EO has great importance in the flavor and food industries because of its unique sensory and quality properties. *Mentha piperita* EO is used as an ingredient in various food and beverage products because it is announced as “generally recognized as safe” by food regulations. Researchers reported that *Mentha piperita* essential oil is used as a flavor agent in commercial products such as chewing gums, candies, chocolates, drinks, herbal tea preparations, cough drops, the tobacco industry, and more [5]. One can understand that *Mentha piperita* EO has high economic and industrial importance since it is involved in a wide variety of consumer products throughout the whole world.

Economically motivated adulteration occurs when fraudsters intentionally substitute an authentic product with cheaper or less valuable materials. The U.S. Food and Drug Administration (FDA) defined “economically motivated adulteration” as the fraudulent, intentional substitution or addition of a substance in a product for the purpose of increasing the apparent value of the product or reducing the cost of its production [6]. Economic importance and wide application area make *Mentha piperita* EO prone to economically motivated adulteration. In this context, the determination of the adulterants in the *Mentha piperita* EO is of great importance in order to maintain and ensure the quality of high-valued natural extract. Authenticity control of *Mentha piperita* EO may prevent dishonest trading, exploitation of consumers, deterioration of the authentic product, and food-safety related problems and health risks originated from adulterants.

According to the scientific reports, essentials oils are adulterated using cheaper oils, various diluents, and synthetic chemical flavor compounds [7]. Previous researches reported that frequently used adulterants of *Mentha piperita* EOs were synthetic menthol and essential oil of cheaper *Mentha* species [8]. These kinds of adulteration deteriorate the quality of authentic essential oil, injure the consumers, and causes negative effects on the whole supply chain from producer to the consumer [9]. There is a need for effective methodologies for discrimination of the authentic *Mentha piperita* EO from fraudulent samples or other *Mentha species*. Additionally, adulterants such as synthetic menthol and cheaper *Mentha* species should be detected and quantified by using strong analytical methodologies. 

Quality standards of *Mentha piperita* EO were determined according to the international standard of “Oil of Pepermint (*Mentha* × *piperita* L.)”. The quality of *Mentha piperita* EO was determined on the basis of various test parameters such as relative density, refractive index, optical rotation, miscibility in ethanol, acid value, and chromatographic profile [3]. These analyses are well-known and trustable, but they are time-consuming and may require toxic chemicals. There is a need for new, rapid, easy, robust, low-cost, eco-friendly analytical techniques for the determination of adulterants in *Mentha piperita* EO. 

FTIR spectroscopy is known as a rapid, non-destructive, reliable, effective, and low-cost analytical technique that provides fingerprint information about the chemical structure of materials [10]. FTIR analyses could be performed using no or minimal sample preparation with very little amounts of essential oils. Previous researches reported the effectiveness of FTIR spectroscopy for quality control of essential oils [9]. The authenticity of natural extracts such as eucalyptus essential oil, lavandin essential oil, pure camellia oil, lavender oil, and geranium oil was successfully determined by using vibrational spectroscopy combined with chemometrics [11,12,13,14,15]. 

There are two primary aims of the current study: 1. To quantify the adulterants *Mentha spicata*, EO and L-menthol in *Mentha piperita* EO, by using ATR-FTIR (attenuated total reflectance-Fourier transform infrared) spectroscopy coupled with multivariate analyses of PLSR (partial least squares regression) and PCR (principal component regression); 2. To distinguish authentic *Mentha piperita* EO from adulterated samples, *Mentha spicata* EO and L-menthol, using ATR-FTIR spectroscopy coupled with HCA (hierarchical cluster analysis) and PCA (principal component analysis)

## 2. Materials and Methods

### 2.1. Devices

Bruker Tensor 27 FTIR spectrometer (Bruker-Germany) was used for the collection of ATR-FTIR measurements. The spectral acquisition was performed in the spectral range of 400 to 4000 cm^−1^. ATR (attenuated total reflectance) unit was used in combination with FTIR spectrometer. *Menhta piperita* EO, *Mentha spicata* EO, and L-menthol were identified by using the library database (ATR-FTIR Complete Library) of FTIR spectrometer.

### 2.2. Samples and Materials

Authentic *Mentha piperita* EO (*n* = 3), *Mentha spicata* EO (*n* = 3) were obtained from the well-known flavor and fragrance companies in Turkey. L-menthol (99%) was bought from a chemist’s shop (Istanbul, Turkey).

### 2.3. Essential Oils and Preparation of Spiked Samples

*Mentha piperita* essential oils are presented as MP1, MP2, and MP3. *Mentha spicata* essential oils are presented as MS1, MS2, and MS3 in the figures and tables. Additionally, L-menthol is presented as LM1, LM2, LM3 in the figures and tables. Spiking of MP1, MP2, and MP3 with Mentha spicata and L-menthol was performed for the concentrations of 0, 1, 2, 4, 8, 16, 32, 64, and 100% (*v*/*v*). We used nine concentration levels to build calibration curves for each analyte. A total of 42 spiked samples were prepared in the scope of this research. Samples were kept at 4 °C in dark vials until spectral acquisition.

### 2.4. ATR-FTIR Measurements

The FTIR spectra of authentic and adulterated EO samples were recorded at the spectral range of 400 to 4000 cm^−1^. 20 µL of data set samples were pipetted with the help of an automatic pipette (20–200 µL). Samples (25 °C) were directly placed on the surface of the diamond ATR crystal. The pipette tip was changed for each different sample. Operation of FTIR spectrometer and data acquisition was accomplished using the software OPUS Version 7.2 (Bruker Gmbh). Spectra were subtracted against the background air spectrum. A total of 16 scans were accumulated for each sample with a spectral resolution of 4 cm^−1^. Measurements were repeated four times for each sample and the average spectra were obtained using the software OPUS Version 7.2.

### 2.5. Multivariate Analyses

#### 2.5.1. Discrimination of Authentic *Mentha piperita* Essential Oil

Authentic *Mentha piperita* EOs were distinguished from adulterated samples, *Mentha spicata* and L-menthol by application of hierarchical cluster analysis (HCA) to the collected spectral data. The software OPUS Version 7.2 (Bruker, Germany) was employed for HCA. HCA was performed by selection of chemometrics parameters of Ward’s algorithm and Euclidian distance. The spectral region of 4000–400 cm^−1^ was chosen to distinguish authentic *Mentha piperita* Essential Oil from spiked samples, *Mentha spicata* and L-menthol.

#### 2.5.2. Prediction of Mentha spicata and L-Menthol Contents of *Mentha piperita* Essential Oil

*Mentha spicata* and L-Menthol contents of spiked samples were quantified utilizing PLSR (partial least squares regression) and PCR (principal component regression) multivariate analysis. Grams IQ (Galactic Industries Corp, Salem, N.H., USA) software was used to perform PLSR and PCR. Multivariate models (calibration and cross-validation) were built using nine spiking levels of 0, 1, 2, 4, 8, 16, 32, 64, and 100% (*v*/*v*). PLSR and PCR models were built on the basis of first and second degree derivatized FTIR spectra data. Different frequency regions were selected for each adulterant since the spectral range should include information describing the concentration variation of the analyte or other matrix constituents [16]. The current paper utilized frequency ranges of 1694 to 1651 cm^−1^ and 1066 to 1034 cm^−1^ for quantification of *Mentha spicata* EO and L-menthol, respectively.

## 3. Results

### 3.1. Spectral Properties of Mentha piperita Essential Oil

**Figure 1:**
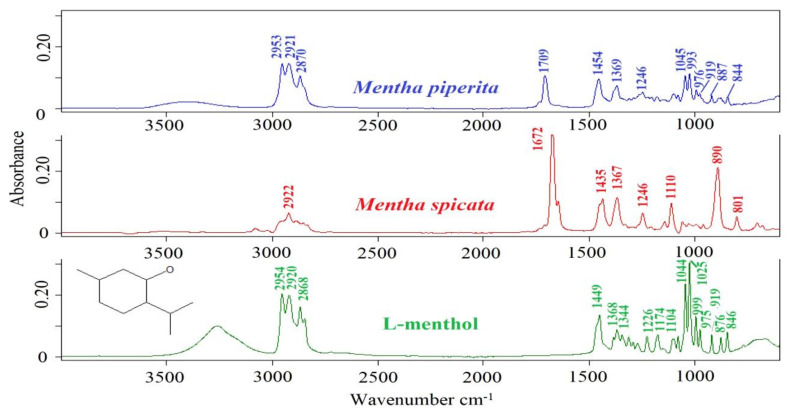
ATR-FTIR spectra of *Mentha piperita* EO, *Mentha spicata* EO and L-menthol in the 4000–400 cm^−1^ spectral region.

ATR-FTIR spectra of *Mentha piperita* EO, *Mentha spicata* EO and L-menthol are presented in Figure 1. ATR-FTIR spectrum of a substance presents the unique chemical composition which is specific to that substance [17]. The effectiveness and capabilities of FTIR spectroscopy provide opportunities to monitor adulterants and suspicious materials in food and beverage matrices. Previous contributions reported that quality control of high-valued natural extracts such as essential oils could be accomplished by using mid-infrared spectroscopy [9]. In the current research, three different *Mentha piperita* essential oils were included in the data set. All of them showed similar spectral characteristics. Prominent spectral bands were observed at 2953, 2921, 2870, 1709, 1454, 1369, 1246, 1045, 993, 976, 919, 887, and 844 cm^−1^. Table 1 presents the spectral ranges and related band assignments of essential oils. Previous studies reported that the spectral ranges of 3100 to 3000 cm^−1^ and 3150 to 3050 cm^−1^ include vibrations arising from the stretching vibrations of C–H groups [18]. The band at 2953 cm^-1^ corresponds to the asymmetric stretching vibrations of –C–H, –CH_3_, and –CH_2_ groups [19]. The peak at 2921 cm^−1^ and 2870 cm^−1^ could be assigned to the –C–H, –CH_2_ asymmetric stretching and –C–H, –CH symmetric stretching vibrations, respectively [19].

The band at 1709 cm^−1^ is due to the –C = O stretching vibrations [19]. The vibrational band at 1454 cm^−1^ could be attributed to deformation vibrations of CH_2_ and CH_3_ groups [20]. The peak at 1369 cm^−1^ corresponds to the stretching vibrations of –C–H and –CH_3_ groups [19]. The spectral band at 1246 cm^−1^ and 1045 cm^−1^ may be assigned to the –C–O stretching vibrations or –CH_2_- deformation vibrations and –C–O stretching vibrations, respectively [19]. The peaks at 993 cm^−1^ and 887 cm^−1^ could be attributed to the (–HC = CH–, trans-) bending vibrations.

### 3.2. Discrimination of Authentic Mentha piperita EOs from Adulterated Samples by HCA and PCA

**Figure 2:**
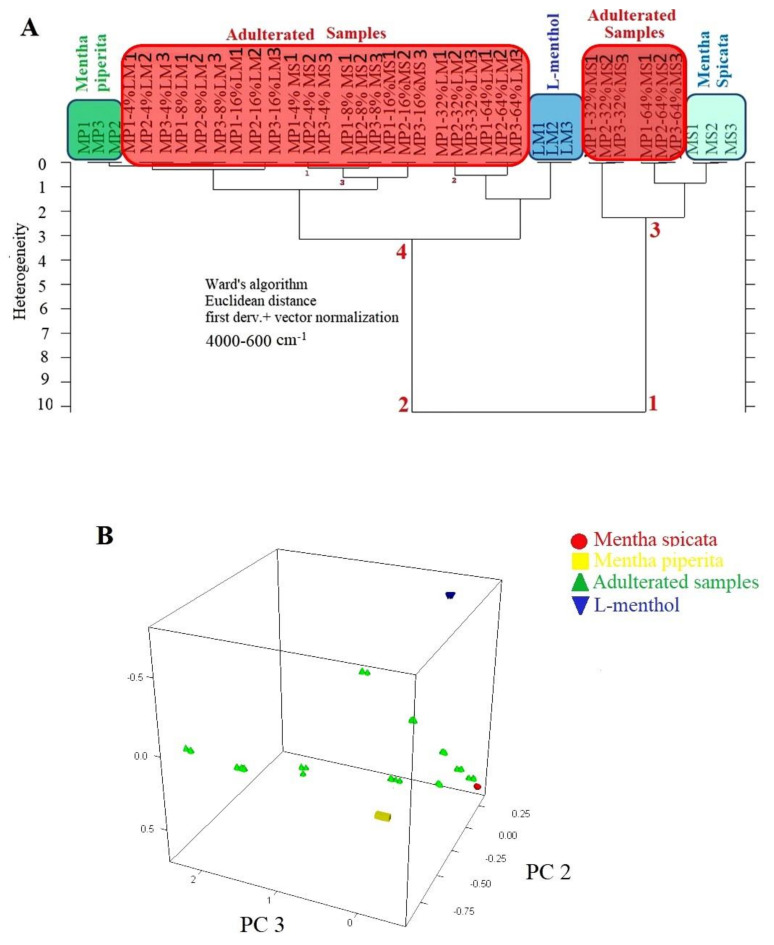
HCA dendrogram of FTIR spectra from *Mentha piperita*, *Mentha spicata*, L-menthol, and adulterated samples (**A**) 3-D PCA plot of FTIR spectra from *Mentha piperita*, *Mentha spicata*, L-menthol, and adulterated samples (**B**).

One of the aims of this study was to distinguish authentic *Mentha piperita* EO from spiked samples, *Mentha spicata* and L-menthol, with the help of discriminative techniques, such as hierarchical cluster analysis (HCA) and principal component analysis (PCA). HCA and PCA analysis was performed by using 1st derivatized FTIR spectra of all samples through Euclidian distance and Ward’s algorithm. A spectral range of 4000 to 600 cm^−1^ was selected for HCA and PCA. Previous Data from several studies reported that the 4000–600 cm^−1^ spectral range included fingerprinting spectral information, which could be used to obtain classification patterns of authentic and counterfeit herbal samples [21]. HCA is an algorithmic approach and it provides an opportunity to observe the classification pattern of authentic and adulterated samples on 2-D dendrogram plots [22]. Dendrograms are composed of branches which include sample sets and sub-sets. Current research utilized Ward’s algorithm to distinguish authentic *Mentha piperita* EOs from other samples. As basic principle, Ward’s algorithm joins at each stage of the cluster pair whose merger minimizes the increase in the total within-group error sum of squares and Ward’s algorithm is not restricted with the general classification problems [23]. Numerous studies employed Ward’s algorithm to determine authentic samples because of the effectiveness of Ward’s algorithm on the discriminative analyses [11,20,24,25,26,27]. It could be concluded from previous studies that the application of HCA coupled with Ward’s algorithm reveals the hidden relationship between investigated samples on the basis of their FTIR spectra. HCA dendrogram of the current study is presented in Figure 2A. As can be seen, *Mentha piperita* samples were clearly distinguished from other *Mentha* species (*Mentha spicata*), L-menthol and adulterated samples. All samples were mainly classified as two main clusters. The right arm of the dendrogram (number 1) included *Mentha spicata* species and *Mentha spicata* adulterated samples in high concentration. The left arm of the dendrogram (number 2) included authentic *Mentha piperita* samples (marked with a green rectangle), L-menthol and Mentha piperita adulterates samples. Adulterated samples were marked by a red rectangle in Figure 2A. HCA not only distinguished the authentic *Mentha piperita* samples, but also presented a classification pattern that revealed the adulteration levels of spiked samples. In other words, the nearest subset to the authentic *Mentha piperita* samples included adulterated samples with 4% spiking level. Precise classification of all samples was obtained with a minimum adulteration level of 4% by using HCA and PCA. 3D-PCA results were presented in Figure 2B. We observed three well-separated clusters and *Mentha piperita* EOs were clearly distinguished from *Mentha spicata*, L-menthol, and adulterated samples. Results from HCA and PCA were coherent with each other. However, HCA provided information about classification patterns and the relationship between the elements of each set and sub-sets.

### 3.3. Prediction of Mentha spicata and L-Menthol Contents of Adulterated Mentha piperita Samples

**Figure 3:**
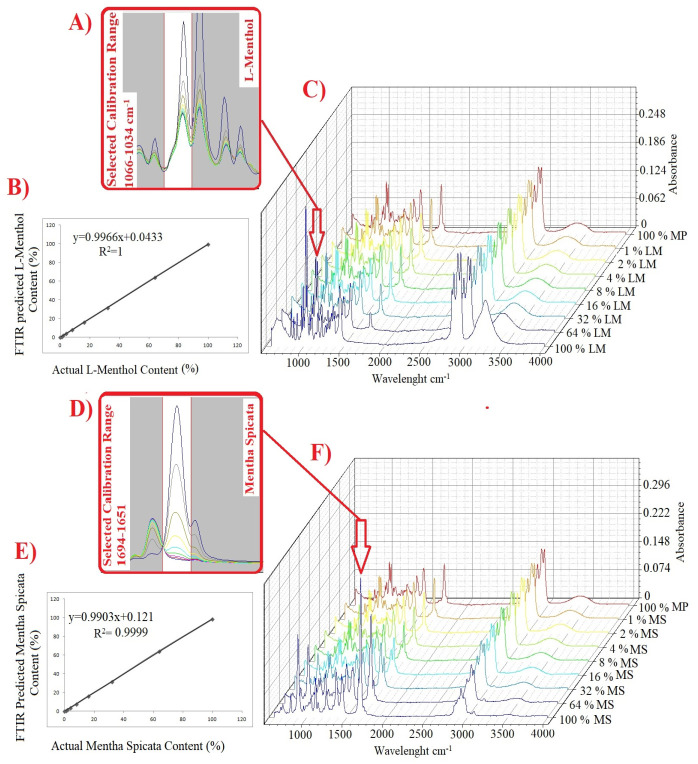
Selected spectral range for quantification L-menthol (**A**) PLSR-raw spectra regression cross-validation plot for L-menthol (**B**) 3-D superimposed FTIR spectra of L-menthol adulterated samples (**C**) Selected spectral range for quantification *Mentha spicata* (**D**) PLSR-raw spectra regression cross-validation plot for *Mentha spicata* (**E**) 3-D superimposed FTIR spectra of *Mentha spicata* adulterated samples (**F**).

The current research used two different multivariate calibration models (PLSR and PCR) for the prediction of *Mentha spicata* and L-menthol contents of adulterated *Menhta piperita* samples. These powerful chemometrics are widely used to detect and quantify suspicious additives or adulterants in the various matrices such as foods, beverages, and essential oil for the sustainability of originality of high-valued products [22]. In the present study, research calibration and cross-validation was carried out for the samples of *Mentha piperita* EO (MP1, MP2, MP3), *Mentha spicata* EO (MS1, MS2, MS3), L-menthol (LM1, LM2, LM3) and adulterated *Mentha piperita* EOs at the concentration levels of 0, 1, 2, 4, 8, 16, 32, 64, 100% (*v*/*v*). Calibration and cross-validation models were developed by using raw, 1st derivative and 2nd derivative spectra of all sample set. These models were built by using specific spectral ranges for each adulterant. Previous contributions reported that the selected spectral range should involve spectral properties describing the concentration variation of the analyte or adulterant [28]. The spectral regions of 1066 to 1034 cm^−1^ and 1694 to 1651 cm^−1^ were chosen to build calibration and cross-validation models which have spectral information to predict L-menthol and *Mentha piperita*, respectively. These spectral regions were presented visually in Figure 3A,D. As can be seen, the absorbance intensity of the spectra increased proportionally with the rise in analyte quantity. Additionally, three dimensional spectra of adulterated samples (0, 1, 2, 4, 8, 16, 32, 64, 100% (*v*/*v*)) were illustrated in Figure 3C,F for adulterants of L-menthol and *Mentha spicata*. 3-D spectra visuals were plotted by using OriginPro software (OriginLab, Northampton, MA, USA). Cross-validation plots, regression equations, and regression coefficient (R^2^) are presented in Figure 3B,E for PLSR-raw spectra of L-menthol and *Mentha spicata*, respectively. The success of PLSR and PCR models were evaluated by the Press, Bias, and SECV values according to the previous reports [28]. “R^2”^ is defined as the regression coefficient and it must be changed between “0” and “1”. When R^2^ equals 1.0, all points lie exactly on a straight line with no scatter. This means that X values let one predict Y values perfectly in the developed regression model [29]. Bias could be defined as the systematic error of the calibration or cross-validation and calculated as the average difference between the reference and predicted values. The standard error of cross-validation (SECV )is calculated as the square root of the residual variance divided by the number of degrees of freedom. The success (performance) of the models were evaluated using SECV (standard error of cross-validation).

(1) $$\mathrm{SECV}\;=\sqrt{\frac{\sum_{i=1}^{m}{{(c}_{i\;}-\;{ĉ}_{i})}^{2}}{m\;-\;2}}$$

where *c_i_* is the reference and *ĉ_i_* is predicted concentration values of ith sample, *m* is the number of samples. The degree of freedom is *m*−2 because when a linear model is assumed, there are only two parameters to be extracted, which are the slope of the actual vs. reference concentration plot and the intercept [30]. 

The results of bias, SECV, and press values are presented in Table 2. Favorable R^2^ values between 0.99 and 1 were obtained for both calibration and cross-validation models of all PLSR and PCR analyses. Then, three latent variables were selected to obtain minimum SECV values in all models. SECV values changed between 0.06 and 2.14. Additionally, bias and press values showed alteration between 0.06 to 1.43 and 0.03 to 41.15, respectively. Results showed that PLSR and PCR models had the considerable capability to quantify *Mentha spicata* and L-menthol in *Mentha piperita* essential oil with high R^2^ values and low SECV and Bias values.

## 4. Discussion

The current study evaluated the capability of rapid, non-destructive, reliable and robust ATR-FTIR spectroscopy coupled with multivariate analyses for detection and quantification of adulterants in *Mentha piperita* EOs. Quality evaluation of *Mentha piperita* EOs is performed using various analysis according to the international standard [3]. Mainly chromatographic analyses are performed to observe the chemical composition of essential oils based on international standard. Chromatographic methods are reliable and accurate, but they are generally laborious, time-consuming, high-cost and more complicated when compared to ATR-FTIR spectroscopy [31]. Results from earlier studies demonstrated the success of ATR-FTIR spectroscopy combined with chemometrics for the determination of the authenticity of essential oils. A previous study determined the geographical origin of Grosso lavandin essential oils of controlled area (GLEOCA) using ATR-FTIR spectroscopy combined with chemometrics and the geographic origin was successfully evaluated using PCA plots [12]. In another study, researchers utilized vibrational spectroscopy with the aid of chemometrics for characterization of high-value geranium oil as an alternative to conventional gas chromatography technique and the content of marker compounds were determined using PLSR multivariate calibration models [15]. In the previous studies, multivariate statistics of PCR and PLSR were successfully employed to calculate main essential oil components such as carvacrol, linalool, myrcene, thymol, etc. Additionally, hierarchical cluster analyses were successfully employed to classify various chamomile oils obtained from different chemotypes and manufacturing processes [32]. Recent evidence suggests that ATR-FTIR spectroscopy can be used as a green, direct, reliable, robust, and cost-effective analytical technique for quality control of essentials oils [20].

To the best of our knowledge, current research is the first attempt for detection and quantification of adulterants of *Mentha spicata* EO and L-menthol in *Mentha piperita* EO using ATR-FTIR spectroscopy coupled with multivariate analyses of PLSR, PCR, HCA, and PCA. Discriminative methods of HCA and PCA were successfully applied to distinguish authentic *Mentha piperita* EO from fraudulent samples on the basis of FTIR spectra. Previous researches mostly dealt with the characterization and antimicrobial properties of *Mentha piperita* EO [4,16,19,20,33,34,35]. Other researches included gas chromatography-mass spectrometry(GC-MS) and PCR (polymerase chain reaction) analysis of essential oils from *Mentha* species [34,36]. Only the studies of Sadowska et al. (2019) and Agatonovic-Kustrin et al. (2020) used FTIR spectroscopy to characterize *Mentha piperita* species and successfully performed band assignments of FTIR spectra from *Mentha piperita* EO. Our FTIR characterization results for *Mentha piperita EO* were quite similar to those they found. Our results showed that HCA and PCA could effectively distinguish authentic *Mentha piperita* EOs from fraudulent samples. In accordance with the present results, previous studies have demonstrated that HCA and PCA can be effectively used for the determination of adulterants in various oil species such as olive oil and mustard oil using ATR-FTIR spectra [37,38].

Additionally, the current study showed that ATR-FTIR spectroscopy coupled with PLSR and PCR had the capability to quantify adulterants (*Mentha spicata* and l-menthol) in *Mentha piperita* EO. These results seem to be consistent with other researches which quantified various adulterants in oil matrices such as hazelnut oil, olive oil, and extra virgin olive oil using vibrational spectroscopy coupled with PLS models [39,40,41].

## 5. Conclusions

The main goal of the current study was to determine the concentrations of *Mentha spicata* EOs and L-menthol in the adulterated *Mentha piperita* EOs using ATR-FTIR spectroscopy coupled with PLSR and PCR. The second aim of this study was to distinguish authentic *Mentha piperita* EOs from adulterated samples, *Mentha spicata* EOs and L-menthol samples.

Adulterant contents (*Mentha spicata* and L-menthol) were successfully calculated using PLSR and PCR models at the concentration range of 0 to 100% (*v*/*v*). SECV values changed between 0.06 and 2.14. Additionally, bias and press values showed alteration between 0.06 and 1.43 and 0.03 and 41.15, respectively. PLSR and PCR showed high accuracy by using raw, 1st derivative and 2nd derivative spectra of all samples. In other words, quite favorable prediction results were obtained utilizing developed all PLSR and PCR models. Additionally, the discriminative analysis was performed to distinguish authentic *Mentha piperita* essential oil from adulterated samples, *Mentha spicata* and L-menthol. A clear classification pattern was observed on 2-D dendrograms. Authentic *Mentha piperita* EOs distinctly classified from adulterated samples and adulterants using cluster analyses of HCA and PCA. These results showed that ATR-FTIR spectroscopy coupled with HCA and PCA could be effectively employed in discrimination of different *Mentha species*, as well as foreign synthetic chemicals, diluents, and low-quality essential oils. The current study performed characterization of *Mentha piperita* EO, *Mentha spicata* EO, and L-menthol by using ATR-FTIR spectroscopy, thus fingerprinting ATR-FTIR data of these samples were contributed to the scientific knowledge.

Taking into account all the mentioned results, FTIR spectroscopy can be considered an appropriate, new, effective, reliable, low-cost and green analytical technique for quality control of essential oils such as *Mentha piperita* EO. Application of the developed models by portable or hand-held options in the whole supply chain may prevent trading of fraudulent essential oil samples.

## Figures and Tables

**Table 1:** Assignment of FTIR spectral bands for essential oils [13,16,18,19,20]. ν—stretching vibrations, δ—deformation vibrations, s—symmetric, as—asymmetric.

IR (cm^−1^)	Assignment
3470	OH
3416	–C = O (overtone) and ν(=C–H, trans-) or ν(–OH)
3100–3000	C–H (Alkene)
3150–3050	C–H (aromatic)
2923, 2875	ν_as_(–C–H, –CH_2_) and ν_s_(–C–H, –CH)
2950	ν_as_(–C–H, –CH_3_, –CH_2_)
1708	ν(–C = O) in acid
1737	ν(–C = O) in ester
1708	ν(–C = O) in acid
1648	ν(–C=C–, cis-) and δ(–OH)
1573	Aromatic ring C = C skeleton
1450 cm	CH_2_ deformation and asymmetrical CH_3_ deformation
1420	C=CH_2_ in-plane deformation vibration
1372/1337	(–C–H, –CH_3_), banding
1285/1244	ν(–C–O) or δ(–CH_2_–)
1124	C-O stretching
1116	ν(–C–O) or δ(–CH_2_–)
1094	ν (–C–O)
1044/1023	ν(–C–O)
991/923	δ(–HC=CH–, trans-) bending out of plane
810	C–H out-of-plane bending
805	δ(–HC=CH–, cis-) bending out of plane δ(–(CH_2_)n–
770	δ(–(CH_2_)n– and –HC=CH– (cis-) bending (rocking)
685	Alkenes

**Table 2:** PLSR and PCR calibration and cross-validation results of raw, first and second derivative FTIR spectra of *Mentha piperita* essential oil.

Sample Codes	Model	Preprocessing	Equation		R^2^		Press	SECV	Bias
			Calibration	Validation	Calibration	Validation			
MS1 MP1 (MS1 adulterated MP1)	PLSR	Raw	y = 1x + 1 × 10^−4^	y = 0.9903x + 0.121	R^2^ = 1	R^2^ = 0.9999	2.15	0.49	0.47
		First derivative.	y = 1x + 0.0001	y = 1.0073x − 0.0816	R^2^ = 1	R^2^ = 0.9999	2.20	0.36	0.34
		Second derivative.	y = 1x + 0.0001	y = 0.9942x + 0.0198	R^2^ = 1	R^2^ = 0.9999	1.83	0.45	0.21
	PCR	Raw	y = 1x + 1 × 10^−4^	y = 0.9918x + 0.1041	R^2^ = 1	R^2^ = 0.9999	1.61	0.42	0.40
		First derivative.	y = 1x + 0.0001	y = 1.0068x − 0.0767	R^2^ = 1	R^2^ = 0.9999	1.03	0.34	0.32
		Second derivative.	y = 1x + 0.0001	y = 0.9939x + 0.022	R^2^ = 1	R^2^ = 0.9999	1.36	0.39	0.22
MS2 MP2 (MS2 adulterated MP2)	PLSR	Raw	y = 1x + 5 × 10^−5^	y = 0.9977x + 0.0314	R^2^ = 1	R^2^ = 1	0.16	0.13	0.11
		First derivative.	y = 1x + 0.0002	y = 0.9914x + 0.0821	R^2^ = 1	R^2^ = 0.9994	5.10	0.75	0.45
		Second derivative.	y = 1x + 0.0003	y = 0.9905x + 0.0618	R^2^ = 1	R^2^ = 0.9998	10.05	1.06	0.35
	PCR	Raw	y = 1x + 4 × 10^−5^	y = 0.9988x + 0.0178	R^2^ = 1	R^2^ = 1	0.07	0.09	0.06
		First derivative.	y = 1x + 0.0002	y = 0.9906x + 0.0901	R^2^ = 1	R^2^ = 0.9993	5.24	0.76	0.48
		Second derivative.	y = 1x + 0.0004	y = 0.9897x + 0.0693	R^2^ = 1	R^2^ = 0.9998	10.15	1.06	0.39
MS3 MP3 (MS3 adulterated MP3)	PLSR	Raw	y = 1x + 4 × 10^−5^	y = 0.9966x + 0.0433	R^2^ = 1	R^2^ = 1	0.29	0.18	0.16
		First derivative.	y = 1x + 4 × 10^−5^	y = 1.0006x − 0.0019	R^2^ = 1	R^2^ = 1	0.03	0.06	0.04
		Second derivative.	y = 1x + 8 × 10^−5^	y = 0.9892x + 0.0713	R^2^ = 1	R^2^ = 0.9998	4.82	0.73	0.41
	PCR	Raw	y = 1x + 4 × 10^−5^	y = 0.9983x + 0.0247	R^2^ = 1	R^2^ = 1	0.12	0.11	0.09
		First derivative.	y = 1x + 4 × 10^−5^	y = 1.0006x − 0.0024	R^2^ = 1	R^2^ = 1	0.03	0.06	0.04
		Second derivative.	y = 1x + 8 × 10^−5^	y = 0.9885x + 0.0764	R^2^ = 1	R^2^ = 0.9998	4.83	0.73	0.43
LM1 MP1 (LM1 adulterated MP1)	PLSR	Raw	y = 1x + 0.0007	y = 0.9903x + 0.121	R^2^ = 1	R^2^ = 0.9994	24.30	1.64	0.14
		First derivative.	y = 0.9998x + 0.0046	y = 0.9746x + 0.299	R^2^ = 0.9998	R^2^ = 0.9991	31.84	1.88	0.98
		Second derivative.	y = 0.9999x + 0.0026	y = 0.9824x + 0.4026	R^2^ = 0.9999	R^2^ = 0.9991	11.03	1.11	0.59
	PCR	Raw	y = 1x + 0.0007	y = 0.9955x + 0.0466	R^2^ = 1	R^2^ = 0.9999	24.30	1.64	0.14
		First derivative.	y = 0.9998x + 0.0046	y = 0.9746x + 0.299	R^2^ = 0.9998	R^2^ = 0.9991	31.84	1.88	0.98
		Second derivative.	y = 0.9999x + 0.0032	y = 1.01x − 0.1024	R^2^ = 0.9999	R^2^ = 0.9992	9.53	1.03	0.53
LM2 MP2 (LM2 adulterated MP2)	PLSR	Raw	y = 0.9999x + 0.0016	y = 0.9955x + 0.0463	R^2^ = 0.9999	R^2^ = 0.9999	22.89	1.60	0.15
		First derivative.	y = 0.9999x + 0.0032	y = 0.9876x + 0.1527	R^2^ = 0.9999	R^2^ = 0.9997	36.95	2.03	0.45
		Second derivative.	y = 0.9999x + 0.0019	y = 0.9679x + 0.3846	R^2^ = 0.9999	R^2^ = 0.9990	18.83	1.45	1.43
	PCR	Raw	y = 0.9999x + 0.0016	y = 0.9954x + 0.0464	R^2^ = 0.9999	R^2^ = 0.9999	10.80	1.09	0.15
		First derivative.	y = 0.9999x + 0.0033	y = 0.9831x + 0.2004	R^2^ = 0.9999	R^2^ = 0.9996	6.82	0.87	0.64
		Second derivative.	y = 0.9999x + 0.0032	y = 1.01x − 0.1024	R^2^ = 0.9999	R^2^ = 0.9992	9.53	1.03	0.70
LM3 MP3 (LM3 adulterated MP3)	PLSR	Raw	y = 0.9999x + 0.0023	y = 0.9955x + 0.0443	R^2^ = 0.9999	R^2^ = 0.9999	41.15	2.14	0.22
		First derivative.	y = 0.9999x + 0.0022	y = 1.0053x − 0.0839	R^2^ = 0.9999	R^2^ = 0.9996	4.09	0.67	0.35
		Second derivative.	y = 0.9999x + 0.0018	y = 0.9952x + 0.0627	R^2^ = 0.9999	R^2^ = 0.9998	8.56	0.98	0.31
	PCR	Raw	y = 0.9999x + 0.0023	y = 0.9955x + 0.0443	R^2^ = 0.9999	R^2^ = 0.9999	41.15	2.14	0.23
		First derivative.	y = 0.9999x + 0.0027	y = 1.0002x − 0.0187	R^2^ = 0.9999	R^2^ = 0.9998	2.34	0.51	0.33
		Second derivative.	y = 0.9999x + 0.0029	y = 0.9929x + 0.0878	R^2^ = 0.9999	R^2^ = 0.9998	3.74	0.64	0.31
